# Matching Unfamiliar Voices to Static and Dynamic Faces: No Evidence for a Dynamic Face Advantage in a Simultaneous Presentation Paradigm

**DOI:** 10.3389/fpsyg.2019.01957

**Published:** 2019-08-23

**Authors:** Sujata M. Huestegge

**Affiliations:** ^1^Department of Special Education and Speech-Language Pathology, University of Würzburg, Würzburg, Germany; ^2^Institute of Voice and Performing Arts, University of Music and Performing Arts Munich, Munich, Germany

**Keywords:** voice-face matching, static vs. dynamic faces, face-voice integration, simultaneous presentation paradigm, person identity processing

## Abstract

Previous research has demonstrated that humans are able to match unfamiliar voices to corresponding faces and vice versa. It has been suggested that this matching ability might be based on common underlying factors that have a characteristic impact on both faces and voices. Some researchers have additionally assumed that dynamic facial information might be especially relevant to successfully match faces to voices. In the present study, static and dynamic face-voice matching ability was compared in a simultaneous presentation paradigm. Additionally, a procedure (matching additionally supported by incidental association learning) was implemented which allowed for reliably excluding participants that did not pay sufficient attention to the task. A comparison of performance between static and dynamic face-voice matching suggested a lack of substantial differences in matching ability, suggesting that dynamic (as opposed to mere static) facial information does not contribute meaningfully to face-voice matching performance. Importantly, this conclusion was not merely derived from the lack of a statistically significant group difference in matching performance (which could principally be explained by assuming low statistical power), but from a Bayesian analysis as well as from an analysis of the 95% confidence interval (CI) of the actual effect size. The extreme border of this CI suggested a maximally plausible dynamic face advantage of less than four percentage points, which was considered way too low to indicate any theoretically meaningful dynamic face advantage. Implications regarding the underlying mechanisms of face-voice matching are discussed.

## Introduction

Current theories of face and voice processing assume strong interactive processing between corresponding visual and auditory input modalities (e.g., Campanella and Belin, 2007). This claim is especially plausible given that similar types of information about a person are processed based on faces and voices, for example, age, gender, ethnicity, masculinity/femininity, health, speech content, personality, emotion etc. A famous example for the combined usage of both streams of information is the McGurk effect, showing that processing different speech input in both channels (e.g., seeing a face uttering/ga/while hearing a voice uttering/ba/) can result in an illusory fused percept (e.g., of hearing/da/, see McGurk and MacDonald, 1976). However, under more natural conditions cross-modal processing interactions do not lead us on the wrong track. Instead, cross-modal information redundancies can, in the absence of experimenters dubbing “wrong” audio tracks to videos, be used to enhance processing of person-related information. For example, it has been shown to be easier to classify a face when it is accompanied with its voice (Joassin et al., 2011). Additionally, a recent cross-modal priming study suggests that face and voice information is already integrated early in the processing stream to enhance recognition (Bülthoff and Newell, 2017). Recent frameworks to account for such effects posit that dynamic information plays a crucial role for the binding of information from several modalities, including the visual (face) and auditory (voice) modalities to enhance person recognition (Yovel and Belin, 2013). In particular, the superior temporal sulcus was assumed to play a major role as a binding hub for dynamic multi-modal information (Yovel and O’Toole, 2016).

A reverse conclusion from this is that faces and voices share common source identity information, thus principally allowing for bidirectional inferences between voices and faces. For example, in a situation where a hotel employee calls our name at the airport to pick us up, we can use his/her voice features to come up with educated guesses as to which one of the faces surrounding us might belong to this voice. Indeed, previous research has demonstrated that the accuracy of matching novel faces to voices is substantially above chance level, thus corroborating the claim that faces and voices share common source identity information.

Lachs (1999) invented a sequential crossmodal matching task which was since then frequently used to study matching of static and dynamic faces to audio samples of voices (e.g., Kamachi et al., 2003; Lachs and Pisoni, 2004a, b; Lander et al., 2007). Such a crossmodal matching task typically consists of four sequential phases per trial: All stimuli (a reference stimulus in one modality prior to two sequentially presented comparison stimuli in the other modality) are presented one after another, followed by a final decision phase (typically a two-alternative forced choice). The sequence of modalities can be varied (visual or auditory reference stimulus first), and the stimuli consisted either of single words (e.g., Lachs, 1999; Lachs and Pisoni, 2004a, b) or sentences (e.g., Krauss et al., 2002; Smith et al., 2016b). Usually, such a design yielded only chance performance when static pictures of faces were used (but see Mavica and Barenholtz, 2013, Experiment 2, for a notable exception), but above-chance performance when dynamic faces were used as stimuli. Based on these findings, it has been argued that temporally dynamic facial information might be a necessary prerequisite for the ability to match faces and voices (e.g., Kamachi et al., 2003).

In the following, an alternative explanation for the discrepancy of results between static and dynamic faces has been proposed. It has been argued that the four-phase paradigm might impose a strong memory load, which could particularly affect the (potentially more demanding) static face condition (Smith et al., 2016b). Indeed, previous literature discussed whether memory-related performance for dynamic faces might be better than for static faces, but corresponding findings in the literature are rather mixed. While some studies reported evidence in favor of an added value of dynamic over mere static facial information (e.g., Thornton and Kourtzi, 2002; see also Dobs et al., 2018, for a recent review), corresponding evidence appears to rely on specific situational demands and parameters of interest (see also Knappmeyer et al., 2003, for a discussion). For example, Christie and Bruce (1998) found an advantage for static over dynamic faces during learning while there was an advantage for dynamic faces at test. Lander and Chuang (2005) showed a benefit for dynamic faces, but their procedure involved the recognition of familiar faces only. Any advantage for dynamic faces – if present – probably results from the presence of more person-related cues, but under some circumstances dynamic information may also hinder face processing (see O’Toole et al., 2002, for an elaborate discussion of potential mechanisms).

Importantly, however, studies using *simultaneous* presentation of at least the comparison stimuli revealed clear above-chance matching performance for static faces (Krauss et al., 2002; Mavica and Barenholtz, 2013; Smith et al., 2016b, Experiment 3). Among these studies, Krauss et al. (2002) presented whole bodies of persons (instead of faces), and only Mavica and Barenholtz (2013, Experiment 1) used a completely simultaneous setting, in which the voice stimulus and two visual face stimuli were presented at once.

Still another matching paradigm involves the sequential presentation of two face-voice pairs, after which participants are asked to directly indicate in which of the two pairs the face matched the voice (same-different procedure, Smith et al., 2016a). This paradigm also revealed above-chance matching performance. Following up on this, Smith et al. (2018) confirmed that any delay between the presentation of the voice and the static face yields matching performance at chance level only. Recently, Stevenage et al. (2017) used a simultaneous same/different matching task and showed above-chance performance for matching voices to static faces. Additionally, they showed that the distinctiveness of the speaker’s voice increased matching performance. Table 1 presents an overview of these previous methodologies.

**TABLE 1:** Overview of previous experimental procedures in face-voice matching studies.

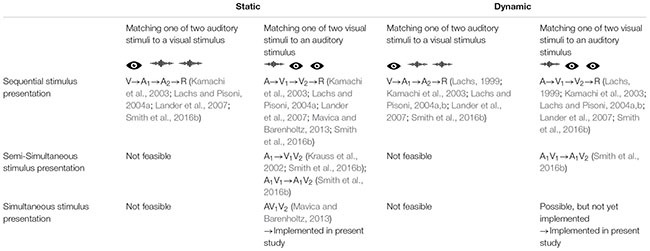

*V = Visual Stimulus (Face); A = Auditory Stimulus (Voice); R = Response in the absence of stimuli.*

In the present study, an experiment involving both static and dynamic faces under optimal (i.e., simultaneous) and comparable conditions was designed. Crucially, both conditions were implemented in a completely simultaneous paradigm in which a voice is accompanied with the display of either two static faces (as in Mavica and Barenholtz, 2013, Experiment 1), or with two dynamic faces, the latter representing a condition not reported until now, even though such a condition more realistically captures matching demands in daily life situations, such as those referred to in the beginning. Participants indicated via a left/right key press which of the two (left/right) faces matches the heard voice while all relevant information is still present (thereby preventing any delay between stimulation and response that was still present in many previous studies). Of course, it is not feasible to implement a modality-reversed condition (matching one face to two simultaneously presented audio files) in such a simultaneous matching paradigm.

Taken together, there is common agreement that dynamic face information can be matched to voices in typical research paradigms that were previously used. Furthermore, there is also some empirical support for the ability to match static faces to voices, but the research so far might be taken as an indication that dynamic face-voice matching abilities are superior to static face-voice matching abilities. In the present study, the aim was to replicate previous findings showing the ability to match dynamic faces to voices, but by using a novel, simultaneous presentation paradigm similar to the one used in previous research for static faces (Mavica and Barenholtz, 2013, Experiment 1). In the same paradigm, the ability to match both static and dynamic faces to voices was tested in order to add further empirical evidence to the conflicting results in previous research, and to compare static and dynamic face-voice matching performance. A careful interpretation of the previous research methodologies suggests that simultaneous presentation should be especially advantageous for finding above-chance matching performance.

Importantly, it was additionally reasoned that one potentially crucial issue in face-voice matching experiments can be the participant’s motivation to comply with the task. That is, some participants may not be sufficiently motivated to actually try to match faces and voices, resulting in matching performance that does not differ significantly from chance level. These participants may sometimes (but not always) be characterized – and therefore identified – by particularly fast RTs or schematic response behavior (e.g., always pressing the same key or two keys in constant alternation). However, it nevertheless appears difficult to finally decide whether a participant had chance-level performance simply because he/she had weak face-voice matching abilities, or whether he/she did not comply with the instructions (or sufficiently attend to the task) in the first place (and should therefore be discarded from the analysis). Unfortunately, in the crucial comparison between static and dynamic face matching performance this can be a quite serious issue, since an unequal number of such non-complying participants between conditions can yield serious performance difference artifacts between groups. Thus, an incidental learning design was utilized to be better able to control for participants that do not sufficiently pay attention to the task.

In the present specific design, another factor was therefore introduced (beside face-voice matching ability) that is also suited to support matching performance based on incidental association learning. Specifically, participants repeatedly encountered the same stimuli across two-choice matching trials with one correct matching option in each trial. This should eventually support an attentive participant’s performance, irrespective of his/her actual face-voice matching abilities. Consider, for example, a Trial 1 in which Voice A is presented alongside Face X and Face Y (i.e., either X or Y must belong to A). If in a later trial (e.g., Trial 5) Voice A is presented again, but with Face Y and Face Z, participants could principally conclude that Face Y must belong to Voice A (since one option must be right, but Z cannot be a correct option since it was not presented in Trial 1). Overall, this should support matching performance, even though only to a limited extent, as participants are clearly not able to memorize all previous combinations and draw corresponding conclusions. Since the presence of face-voice matching abilities is already well established in the literature, it was reasoned that it is not a severe problem that this procedure does not allow for a dissection of the final performance into an incidental learning portion and face-voice matching ability portion. Instead, this procedure should allow for judging whether participants pay attention to the task at hand and generally try to comply with instructions, assuming that any attentive participant should benefit from the incidental learning cues to achieve above-chance performance. Thus, all participants that do not show above chance performance could be excluded (here: equivalent to less than 100 correct trials out of 172, see method section for details), assuming that these participants did not sufficiently pay attention to the task demands. Eventually, this should help to only include attentive participants in both groups and thereby contribute to a better comparability between groups.

## Materials And Methods

### Participants

Sample size considerations were based on a previous experiment that involved a similar procedure (regarding the static face condition), namely Experiment 1a in Mavica and Barenholtz (2013). Specifically, they also presented two (static) faces (including contextual features such as hair etc.) while full auditory sentences were played back. Furthermore, they also utilized a (roughly) comparable number of trials. A power analysis based on their effect size (Cohen’s *d* = 1.91), an alpha level of 5% (one-tailed) and a power of 95% resulted in a required sample size of 5 participants. Since I was interested in a potential *difference* in matching performance between static and dynamic stimuli, it was decided to increase the sample size considerably to be able to derive precise interval estimates for this particular difference as a basis for solid theoretical conclusions.

Originally, the aim was to test 48 participants in the experiment (24 in the dynamic faces group and 24 in the static faces group). Participants were randomly assigned to either of the two conditions (static vs. dynamic faces). All participants had normal (self-reported) hearing and normal or corrected-to-normal vision. They received monetary reimbursement (or a small present) for participation. All participants gave informed consent and were treated in accordance with the Declaration of Helsinki (the study is considered exempt from a full ethic vote procedure from the local ethics committee).

In the dynamic group, 5 participants of 24 showed performance (in terms of errors) that was not significantly different from chance level (note that 4 of them were participants with the shortest mean RTs within their group, further corroborating the assumption that these participants did not pay close attention to the task). Thus, the sample was filled up with 5 new participants (performance of one participant did again not differ significantly from chance). In the static group, performance of one of the 24 participants did not differ significantly from chance (I decided against replacing this single data set with a new participant). Thus, each group finally consisted of 23 participants with above-chance performance (i.e., indicating attention to task, see above). Note that the decision to re-test participants based on low performance actually worked in favor of the measured performance in the *dynamic* condition, as more participants were re-tested due to low initial performance in this particular group.

In sum, the sample in the static group had a mean age of 25 years (*SD* = 4.6, range: 20–36 years, 4 left-handed, 7 male), while the dynamic group had a mean age of 24 years (*SD* = 4.2, range: 19–33 years, 2 left-handed, 6 male). The age difference between groups was not statistically significant, *t* < 1.

### Stimuli and Apparatus

**FIGURE 1:**
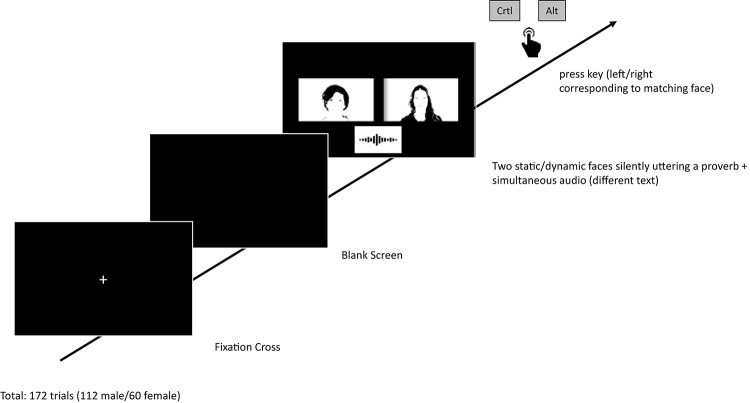
Schematic trial sequence. The fixation cross was presented for 316 ms, followed by a briefly presented blank screen (16 ms). Stimuli were presented as long as participants terminated the trial with a left or right key press response (stimuli were looped to ensure that visual and auditory information was consistently present until responding).

Participants were seated in a dimly lit (and sound-isolated) room about 60 cm in front of a TFT computer screen (24″., resolution: 1920 × 1200 pixels, running at 60 Hz) with a standard computer keyboard in front of them. Two keyboard keys (left Ctrl, Alt) served as response keys to indicate the visually presented face that matches the voice (displayed on the left/right of the screen). The stimuli were based on original video and audio recordings of 8 male and 6 female speakers (professional Caucasian theater actors; age range: 29–53 years, dialect-free native speakers, neutral facial expression, unambiguous male/female face and voice). Since the present study aimed at natural visual face presentation, the videos also showed the hair and upper torso of the actors. Note that stimulus gender was not a factor in the present design as previous research reported a lack of any effects of stimulus (or participant) gender (Mavica and Barenholtz, 2013). However, stimulus gender was relevant for the methodology since it should be ensured that all stimuli in each trial (i.e., the two faces on the screen and the simultaneously presented voice) were always of the same gender. Including young and elderly persons as stimuli was deliberately avoided to minimize the potential of age serving as a strong cue for matching performance. Stimuli were based on an audio file with a mean duration of 22.1 s (*SD* = 2.9) across all speakers, and represented a standardized short German text (sample from “Der Nordwind und die Sonne,” approx. translation: “the north wind and the sun”). The auditory stimuli were presented via headphones. The sound pressure level was adjusted based on pilot experiments in order to be clearly audible without being considered too loud by sample participants (there was no overlap with actual participants used in the experiment). At the same time, participants either saw two pictures of faces on the left and right side of the screen (static face condition), or two (silent) video clips of faces on the left and right side of the screen (neutrally) uttering a proverb (“aus einer Mücke einen Elefanten machen,” approx. equivalent to “make a mountain out of a molehill,” dynamic face condition). In the dynamic face condition, the proverb was looped, thereby the corresponding moving faces were visible throughout the trial. The mean duration of a single utterance (single loop) amounted to 1.9 s (*SD* = 0.25). The correlation of the models’ speech rate between the (visually presented) proverb and the (auditorily presented) text amounted to *r* = 0.70, *p* = 0.006, indicating that fast proverbs speakers also tended to be fast text speakers. The looped videos were edited using the software Pinnacle (Version 21.0 Ultimate, CorelDRAW^®^, Ottawa, ON, Canada), while the experiment was programmed using the software Presentation (Version 19.0, Neurobehavioral Systems, Inc., Berkeley, CA). The latter was also used to record the responses (key presses) and response times (RTs). The image/video combinations were displayed (presented side-by-side adjacent to the left and right of the screen center (as depicted in Figure 1), each picture/video subtending 23° horizontally and 13° vertically) on black background.

### Procedure

The experiment started with an instruction screen. In each trial, after a brief presentation of a central fixation cross, participants were asked to attend to the heard voice and the two (static or dynamic) faces and to press the (left/right) key corresponding to the respective face that, according to the participant’s suggestion, best matched the voice. The stimuli were presented as long as participants gave their response; speed was not emphasized in the instruction. After each response, presentation of the stimuli finished and the next trial started (see Figure 1). Participants in each group completed 172 trials. Eight male speakers, combined with all respective other male models, resulted in 56 trial combinations. Each combination was presented twice (switching left and right positions), resulting in 112 male trials altogether. The same was done with the female models, resulting in 30 × 2 = 60 female trials. Male and female trial combinations were presented in random order. No performance feedback was given. After completing the matching task, participants filled out a qualitative post-experiment enquiry, involving questions regarding the basis on which they derived their choices.

## Results

### Matching Performance

**FIGURE 2:**
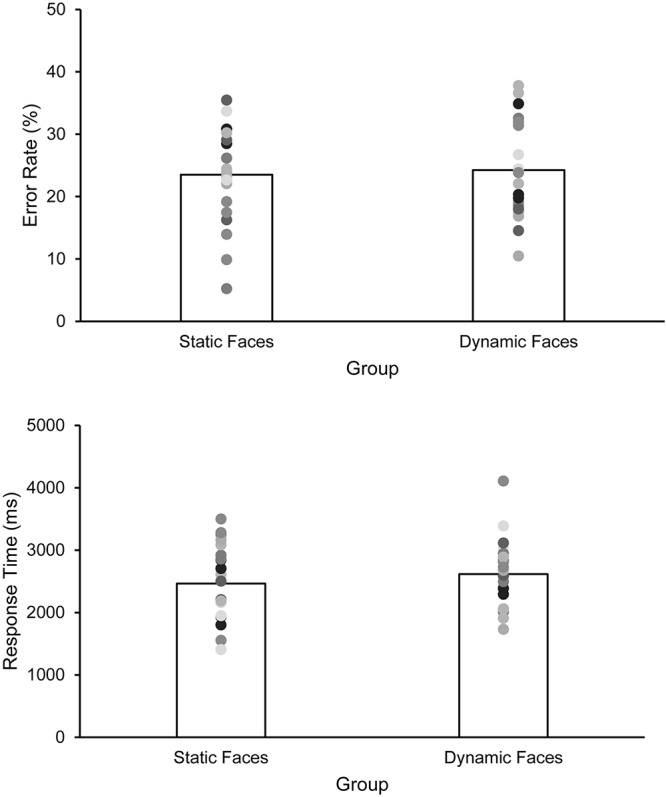
Matching performance (**upper panel**, error rates in %) and RTs (**lower panel**, in ms) as a function of group (static vs. dynamic faces). Bars indicate the arithmetic means, dots represent individual data points. Note, however, that instructions did not emphasize response speed in the first place.

The error rate amounted to 23.51% (*SD* = 7.52) in the static group and 24.27% (*SD* = 7.53) in the dynamic group, *t*(44) = 0.342, *p* = 0.734, *d* = 0.101 (Figure 2). The 95% *CI* for the mean group difference of 0.76% ranged from –3.7 to 5.2%. Even at the extreme *CI* border (–3.7%) this difference clearly speaks against the assumption that additional dynamic (in contrast to mere static) information substantially contributed to matching performance (see Discussion for an additional corresponding Bayesian analysis).

### Response Times

RTs < 600 ms (equivalent to two trials overall) as well as RTs > 6000 ms (equivalent to 8.3% of trials; static group: 6.27%, *SD* = 8.4, range: 0–30%; dynamic group: 10.36%, *SD* = 11.59, range: 0–38%, *t*(44) = 1.37, *p* = 0.179) were removed as outliers from RT analyses. Mean RTs were 2465 ms (*SD* = 576) in the static group and 2617 ms (*SD* = 530) in the dynamic group, *t*(44) = 0.935, *p* = 0.355, *d* = 0.275.^1^ Note that including all data (i.e., including all outliers) did not change this pattern of results (static: 2803 ms, *SD* = 911; dynamic: 3209 ms, *SD* = 1068; *t*(44) = 1.39, *p* = 0.172, *d* = 0.409). In the static group, error rates negatively correlated with RTs (*r* = –0.668, *p* < 0.001), whereas this was not the case in the dynamic group (*r* = 0.248, *p* = 0.254). The difference between these two correlations (based on the procedure described in Howell, 2007, p. 259f) was statistically significant, *p* < 0.05.

### Item-Based Analyses

As an additional *post hoc* analysis, I also analyzed individual voice stimuli regarding their associated matching performance. There was no significant between-group RT difference for any of the individual voice stimuli, all *p*s > 0.05. Regarding error rates, two female stimuli (out of all 14 stimuli) significantly differed in error rates between groups (both *p*s between 0.01 and 0.05), but these two differences pointed into opposite directions. Overall, performance for individual stimuli was therefore quite comparable between the two groups.

There were significant matching performance differences (evidenced by error rates) between the individual male stimuli, both in the static, *F*(7, 154) = 9.187, *p* < 0.001, η_*p*_^2^ = 0.295, and in the dynamic, *F*(7, 154) = 5.244, *p* < 0.001, η_*p*_^2^ = 0.192, condition. This was also found for the female stimuli, *F*(5, 110) = 2.940, *p* = 0.028, η_*p*_^2^ = 0.118, *F*(5, 110) = 5.684, *p* = 0.001, η_*p*_^2^ = 0.205, respectively (see following analysis for more details on range and individual means). These effects indicate that some voices were easier to match with faces than others.

Error rates for the voice stimuli were significantly correlated across groups, *r* = 0.667, *p* = 0.009, suggesting that voice stimuli that were (relatively) easy to match with static faces were also easy to match with dynamic faces (see Table 2).

**TABLE 2:** Static and dynamic matching accuracy for individual stimulus models.

**Stimulus Gender**	**Stimulus ID**	**Stimulus Age**	**Matching Errors (Static Group)**	**Matching Errors (Dynamic Group)**
Male	1	29	29%	23%
	2	46	20%	17%
	3	34	13%	19%
	4	46	9%	9%
	5	41	23%	29%
	6	53	9%	16%
	7	35	26%	18%
	8	36	32%	28%
Female	1	35	27%	17%
	2	37	36%	31%
	3	32	31%	31%
	4	28	19%	39%
	5	47	26%	31%
	6	34	38%	49%

A qualitative analysis of these performance indices in the context of the *age of the stimulus models* (see Table 2) did not reveal any clear evidence that stimulus models at the edges of their respective age distribution were particularly easy to match (which would indicate that age served as a central cue for matching abilities): While the oldest male stimulus model was indeed associated with best performance in the static group, this was no longer the case in the dynamic group. In turn, the youngest male stimulus model was actually associated with the *worst* performance in the static group (and still among the worst in the dynamic group). For the female stimulus models, there was also no clear indication for age as a central cue for matching performance: The oldest female stimulus model was neither associated with best performance in the static nor in the dynamic group. While the youngest stimulus model was indeed associated with best performance in the static condition, she was associated with relatively low performance in the dynamic condition (where the best performance was associated with a stimulus model whose age was exactly the mean age of the female stimuli). Taken together, there was no consistent indication that age was a highly informative cue for matching abilities (note, however, that the number of stimuli was too low to conduct meaningful quantitative, statistical analyses).

### Initial Matching Performance (Without Incidental Learning)

Even though the present design is not suited to reliably assess matching performance without the additional impact of incidental learning of matching patterns (see section “Introduction”), I additionally analyzed only the first trial of each participant (i.e., each participant contributed only one single trial to the analysis) to assess matching accuracy without any contribution of incidental association learning. The latter may occur, for example, due to the following scenario: When Trial 1 involves Voice A alongside Faces X and Y, either X or Y must belong to A. If in a later trial Voice A is presented again, but with Faces Y and Z, participants could principally conclude that Face Y *must* belong to Voice A (see section “Introduction” for more details). Thus, when only the first trial for each participant is analyzed, such incidental learning can be excluded (although at the expense of the reliability of performance estimates per participant). When combining both groups, a one-sided binomial test revealed statistically significant above-chance matching performance (*N* = 46, observed proportion: 65% correct, 35% incorrect, *p* = 0.027). Of course, corresponding group-wise analyses suffer from severe power limitations. Nevertheless, these group-wise analyses still revealed above-chance performance at least in the dynamic group (*N* = 23, 74% correct, *p* = 0.017), but not in the static group (*N* = 23, 57% correct, *p* > 0.05). Overall, this analysis further confirms the central assumption that participants were able to match faces and voices (even in the absence of any support based on incidental association learning), thereby replicating previous studies (see section “Introduction”). Note, however, that this analysis – due to its compromised reliability (and thus statistical power) – does not allow for any conclusions regarding between-group differences in this type of analysis.

### Learning of Face-Voice Matching

**FIGURE 3:**
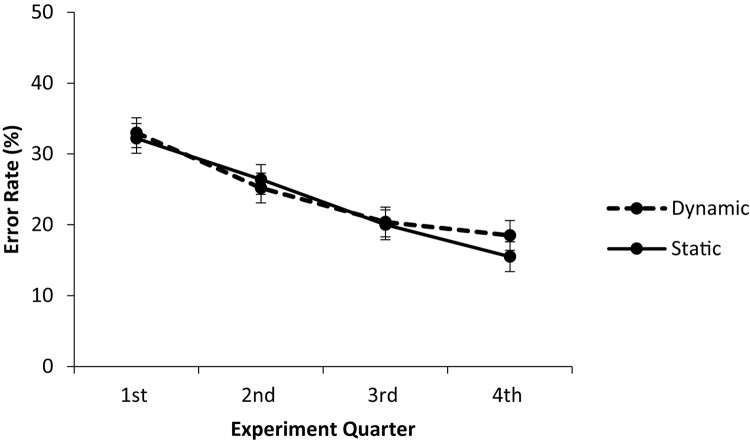
Development of face-voice matching performance in the static and dynamic group over the course of the experiment.

To assess the development of matching performance in the static and dynamic groups over the course of the experiment, error rates were additionally analyzed as a function of experiment quarter (see Figure 3).

A corresponding ANOVA with the within-subject factor experiment quarter and the between-subject factor group revealed a significant main effect of experiment quarter, *F*(3, 132) = 31.93, *p* < 0.001, η_*p*_^2^ = 0.421, but neither a significant main effect of group nor a significant interaction, *F*s < 1, suggesting that learning rate did not significantly differ between the static group and the dynamic group.

### Qualitative Questionnaire Analysis

Finally, the participants’ verbal answers to the question regarding their subjectively experienced criteria to complete the matching decisions were analyzed qualitatively. Specifically, I was interested in the participants’ awareness regarding the incidental learning support inherent in the present design. However, only 2 participants in the static group and 3 participants in the dynamic group mentioned that they somehow made use of the repeated presentation of faces and voices (in different combinations) as a cue to improve performance.

## Discussion

Taken together, the present study – which utilized a novel procedure to ensure attention to task based on implicit learning support – yielded no evidence for any meaningful incremental usability of dynamic information in face-voice matching. Note that this conclusion was not merely derived from the lack of a statistically significant group difference in matching performance (which could principally be explained by low statistical power), but from an analysis of the 95% confidence interval (CI) of the actual effect size. The extreme border of this CI suggested a maximally plausible dynamic face advantage of 3.7 percentage points, which was considered way too low to indicate any theoretically meaningful dynamic face advantage. To back up this conclusion, an additional Bayesian analysis (using the open source software JASP with default priors and by specifying a dynamic face advantage as alternative hypothesis) resulted in BF_01_ = 4.291, translating into reasonable evidence for the null hypothesis (i.e., the data are more than 4× more likely to be observed under the null hypothesis than under the alternative hypothesis).

This result therefore challenges earlier assumptions postulating that dynamic facial cues are of particular importance for matching faces to voices: Specifically, it has been argued that temporally dynamic facial information might be a necessary prerequisite for the ability to match faces and voices (e.g., Kamachi et al., 2003). The present study is also the first to compare static and dynamic faces in an ideal situation for face-voice matching by simultaneously presenting all stimuli at the same time to reduce any memory-related additional demands (for sequential paradigms see: Lachs, 1999; Kamachi et al., 2003; Lachs and Pisoni, 2004a, b; Lander et al., 2007). Note that a methodological strength of the present incidental learning approach is that a way to control for participants lacking sufficient attention to task demands was found, a phenomenon that has the potential for yielding strong artifacts in comparisons of static and dynamic face-to-voice matching performance (i.e., whenever the occurrence of such participants slightly differs between groups and therefore creates spurious group differences). Interestingly, this procedure initially led to more exclusions of participants in the dynamic condition, not in the static condition. Thus, whatever the information used by participants to complete the task (e.g., hormone levels expressed in both face and voice etc.), it should mainly be already present in the static face, and only to a negligible extent in dynamic (speaking-related) information.

Previous studies that have not revealed above-chance static face-voice matching ability were likely hampered by the strong memory load associated with sequential stimulus presentation paradigms, which could particularly affect the (potentially more demanding) static face condition (Smith et al., 2016b). This reasoning is also in line with a previous study suggesting that face matching performance for dynamic faces is better than for static faces (Thornton and Kourtzi, 2002), but it should be noted that, overall, the literature revealed rather mixed findings regarding a general superiority of dynamic over static face processing (see section “Introduction”). Notably, however, face-voice matching studies that used a simultaneous presentation of at least the comparison stimuli revealed clear above-chance matching performance for static face stimuli (Krauss et al., 2002; Mavica and Barenholtz, 2013; Smith et al., 2016b, Experiment 3), which is in line with the observations in the present study.

The present results are also important as they replicate previous reports of successful face-voice matching ability with both dynamic and static faces, but with a different set of stimuli. Many of the previous reports only rely on a few sets of selected stimuli, and it is therefore important to replicate corresponding effects using new sets of faces and voices.

However, the present approach also has several potential limitations which should be discussed. First, the incidental learning procedure does not provide a separate estimate of face-voice matching ability (i.e., unconfounded with a general ability to implicit learn cross-modal stimulus associations). At first sight, one might therefore challenge the assumption that face-voice matching ability substantially contributes to performance in the present task in the first place. However, this argument is not compatible with the analysis of only the first trial in each participant, which (when taking both groups into account) clearly revealed significant matching abilities in the present sample of participants. Also note that there was no indication of a ceiling effect in the performance of the participants, and there was a reasonable extent of between-participants variability. Again, these observations further support the claim that incidental learning did not override any face-voice-matching-related effects.

Another potential issue is that there was no statistically significant evidence for static face-voice matching ability when analyzing only the first trials in these 23 participants (as opposed to a significant corresponding effect in the dynamic condition). At first sight, this observation may seem to corroborate suggestions from previous research that matching ability is enhanced for dynamic stimuli, since these yielded significant above-chance matching ability in the first-trial analysis. However, several observations speak against this conclusion. First, full performance (across all trials) was highly comparable between both groups. Thus, the only way to reconcile this latter observation with the assumption of lower static (vs. dynamic) face-voice matching abilities would be to assume that incidental association learning between voices and static faces is much easier than for dynamic faces (in order to compensate for any reduced static face-voice matching ability to eventually come up with similar overall performance in the full analysis including all trials). However, to my knowledge there is neither a plausible explanation (as dynamic face stimuli should, if anything, boost attention to the stimulus as a prerequisite for learning) nor a corresponding finding from the association learning literature that may substantiate such a claim (and note that participants were randomly assigned to groups). Most importantly, the present data showed that the learning rates for face-voice matching over the course of the experiment were highly similar for the static and dynamic faces groups. Taken together, the most likely explanation for the lack of a significant face-voice matching effect in the static group using the first trial per participant only is simply a lack statistical power for this particular analysis, especially as the direction of the effect in the static group pointed into the expected direction. Finally, the assumption of significant static face-voice matching ability is also confirmed by previous studies demonstrating corresponding effects (Krauss et al., 2002; Mavica and Barenholtz, 2013, Experiment 2; Smith et al., 2016b, Experiment 3), and some studies suggested that the absence of strong memory demands (as realized in the present design) is especially beneficial for yielding above-chance static face-voice matching ability (Smith et al., 2016a, 2018; Stevenage et al., 2017). Taken together, there is no substantial reason to doubt the presence of face-voice matching abilities in both groups of participants.

In the following, one final potential limitation of the present study should be discussed in more detail. Specifically, one might argue that the present procedure (simultaneous presentation of two visual static/dynamic stimuli along with auditory speech) might represent an *a priori* disadvantage for the dynamic condition due to potential interference phenomena (see Huestegge and Raettig, 2019; Huestegge et al., 2019, for underlying cognitive mechanisms of such interference phenomena). In particular, different text material was used for the two input channels in the dynamic condition, resulting in an audio-visual mismatch between the lip movements of the actors and the auditory text (i.e., seen and heard speech). This may have created interference or increased cognitive load such that performance was negatively affected by increased task demands in the dynamic condition. Of course, it cannot completely be ruled out that this methodological feature of the present study has contributed to the results. Nevertheless, several considerations speak against this alternative explanation. First, it is important to note that different text material across the two input channels was deliberately used to control for potential confounds. Using the same text material would have allowed the participants to solve the matching task simply based on the amount of synchrony between the visual and auditory input (this would even hold when different takes were used to synchronize stimuli in which the face and voice actually belong to the same person). Another possibility could be to match the auditory text to a dynamic visual face that does not utter text. However, beside the fact that this would also introduce cross-channel perceptual interference (between seeing a face that does not speak and spoken auditory text) this would *a priori* exclude the possibility that participants solve the matching task based on dynamic information that is especially relevant during speaking. Finally, it was considered unlikely that the mismatch between heard voices and visual lip movements created substantial interference for the participants in the first place. All participants were German, who are culturally accustomed to watching synchronized movies in German television (thus, cross-modal asynchrony between lip movements and heard text is a standard phenomenon which is usually not experienced as substantial cognitive load). Second, it is reasonable to assume that participants got used to any perceived cross-modal asynchrony across the many trials of the experiment, especially as the instructions/task did not require participants to focus on speech content at all (thus creating no need to resolve any potential cross-modal interference). Finally, none of the participants explicitly mentioned any perceived difficulty associated with the dynamic condition in the free follow-up interview after the experiment, in which they were explicitly asked to report anything they noticed regarding the experiment. To conclude, it can be considered unlikely that cross-modal interference based on a mismatch between head voice and visual face substantially increased task difficulty selectively in the dynamic condition, especially since the static condition also involved a mismatch (i.e., the lips of the static faces do not move despite the presentation of spoken text). Conversely, I firmly believe that comparing static and dynamic visual faces in a simultaneous presentation paradigm for the first time allows for a fair comparison of static and dynamic conditions as participants do not have to rely on memory, which is already well-known to substantially compromise any interpretation of face-voice matching performance (Smith et al., 2016a, 2018; Stevenage et al., 2017).

Nevertheless, further research is clearly needed that should demonstrate static face-voice matching ability with the present stimulus set without using an incidental association learning support, for example, by using fewer trials (without stimulus repetitions), a larger set of stimulus models, and a larger number of participants to counteract any potential compromise in statistical power. The present results (as well as previous studies, see above) strongly suggest that significant matching performance should be observed under these conditions.

Another potential limitation of the present study that should be discussed is the variation of stimulus model age, which might potentially serve as a matching cue. In general, both voices and faces change with age, so that age can principally be used as a cue to match unknown faces and voices. However, most of the changes in voice characteristics occur outside the age range of the stimulus models used in the present study. For example, a study by Lortie et al. (2015) compared several voice-related parameters between different age groups, including a young age group (20–39 years) and a middle-aged age group (40–65 years). However, there was no significant difference between these two age groups in terms of voice jitter, shimmer, and amplitude (dB). While there was a significant difference between these groups with respect to voice-related signal-to-noise ratio, it has to be kept in mind that the age range of these two groups was substantially larger than the age range of the stimulus models utilized in the present study. Another study by Nishio and Niimi (2008) specifically focused on changes in fundamental frequency as a function of age and found no significant changes for males and females in their 30, 40, and 50 s. Other studies utilized regression approaches covering voice feature changes across the whole life span. A closer look at the rate of various voice feature changes in the regression plots of these studies also suggests that substantial changes typically occur outside the age range of the stimulus models used in the present study (e.g., Linville, 1996; Stathopoulos et al., 2011). Finally, a study by Hughes and Rhodes (2010) explicitly focused on the ability to assess age based on voice. A central result was that the mean absolute difference between actual age and voice-based age estimation amounted to about 11–17 years in the stimulus age group of 35–55 years, again highlighting that age assessment in this age range is highly unreliable. Taken together, these previous observations are quite in line with the qualitative analysis of the present data, which also revealed no clear evidence for the idea that stimulus age was a useful cue for face-voice matching. Nevertheless, in a future study it would still be advisable to use an even narrower age range for the stimulus models to further minimize any possibility of using perceived age as a cue to increase matching performance.

The present results are overall in line with current theories of face and voice processing that assume a strong interaction between corresponding visual and auditory input modalities (e.g., Campanella and Belin, 2007). The assumption of a strong interaction is especially plausible given that similar types of information about a person are processed based on faces and voices. However, given the overwhelming evidence for matching ability in the literature, more future research should be devoted to address the underlying mechanisms of this ability in terms of the specific sources of the correlates between voice and face features that participants use to match faces and voices (e.g., hormone levels affecting both faces and voices in a predictable manner). Such “concordant information” includes a variety of potential characteristics (see Smith et al., 2016b, for a review). For example, faces and voices both contain information regarding genetic fitness and attractiveness, masculinity/femininity (based on hormone levels), age, health, height, and weight (see also Yehia et al., 1998). The present results suggest that some relevant sources should already affect static face characteristics, and are not predominantly expressed in facial movements while speaking.

Interestingly, Nagrani et al. (2018) recently presented a computer algorithm that was claimed to even exceed face-voice matching abilities of humans, merely based on physical features of (static and dynamic) visual face information and voice audio (not taken from the same video material). Again, this corroborates the common source information hypothesis and opens up interesting technical possibilities of exploring such common features across modalities in technical applications in the near future.

In sum, the present study compared face-voice matching performance between static and dynamic face groups and revealed a lack of theoretically relevant differences in matching ability. Consequently, dynamic (as opposed to mere static) facial information does not appear to contribute substantially to face-voice matching.

## Data Availability

The datasets generated for this study are available on request to the corresponding author.

## Ethics Statement

This study was carried out in accordance with the recommendations of Declaration of Helsinki, with written informed consent from all subjects. All subjects gave written informed consent in accordance with the Declaration of Helsinki. The protocol is considered exempt from a full ethic vote of the Ethics Committee of the Institute of Psychology, Würzburg, Germany.

## Author Contributions

SH designed, ran, and analyzed the study and also wrote the manuscript.

## Conflict of Interest Statement

The author declares that the research was conducted in the absence of any commercial or financial relationships that could be construed as a potential conflict of interest.

## Footnotes

1 Upon a reviewer’s request, I also analyzed matching performance and response times by additionally including stimulus gender as a within-subject factor and (participant’s) age as a covariate using a mixed ANCOVA. However, for both dependent variables there were neither significant main effects of stimulus gender and group, nor significant effects of the covariate age, nor any significant interactions (all *p*s > 0.05).
